# Dermatofibrosarcoma Protuberans Arising From a Chronic Wound in the Left Shoulder: A Case Report

**DOI:** 10.7759/cureus.55638

**Published:** 2024-03-06

**Authors:** Nancy Zeaiter, Charbel B Aoun, Joseph Sfeir, Maher Ghandour, Walid Hreibe

**Affiliations:** 1 Plastic and Reconstructive Surgery, Lebanese University, Beirut, LBN; 2 Plastic and Reconstructive Surgery, Al-Zahraa Hospital University Medical Center, Beirut, LBN; 3 Orthopedics, CHU Grenoble Alpes, Grenoble, FRA

**Keywords:** soft-tissue sarcoma, nonhealing ulcer, histopathology, chronic wound, dermatofibrosarcoma protuberans

## Abstract

Dermatofibrosarcoma protuberans (DFSP) is a rare, slow-growing soft tissue sarcoma, typically presenting as a cutaneous lesion. However, its occurrence in chronic wounds is infrequently documented, posing diagnostic and therapeutic challenges. This report details the case of a 59-year-old female with no significant medical history, presenting with a chronic, non-healing wound on the left shoulder, persisting for three years. Initially a small nodule, it progressed into an ulcerating lesion. Physical examination revealed a contracted scar with restricted shoulder mobility. After obtaining informed consent, a surgical excision of the lesion was performed by an electrocautery. Histopathology confirmed DFSP, characterized by spindle fibrous cells, with skin ulceration and deep dermal infiltration. A split-thickness skin graft achieved successful closure. This case underscores the importance of considering DFSP in chronic, non-healing wounds. Timely intervention and appropriate surgical management are crucial for favorable outcomes.

## Introduction

Dermatofibrosarcoma protuberans (DFSP) is an uncommon soft tissue sarcoma, primarily affecting the dermis and subcutaneous tissues. It is characterized by its slow growth, low metastatic potential, but relatively high rate of local recurrence [1]. DFSP typically presents as a firm, cutaneous nodule, and its diagnosis is often delayed due to its indolent nature and resemblance to benign skin lesions [2].

The transformation of DFSP from chronic wounds is an extremely rare occurrence, with few documented cases in medical literature. This raises significant clinical interest, as chronic wounds are more commonly associated with conditions like diabetes, venous insufficiency, or pressure ulcers, and not typically with neoplastic transformations [3].

The management of DFSP involves a multidisciplinary approach, with an emphasis on surgical excision with adequate margins to prevent recurrence. Histopathological examination plays a vital role in confirming the diagnosis and guiding treatment strategies [4].

In this paper, we report a unique case of DFSP arising from a chronic wound in a patient with no significant medical history. This case is noteworthy for its unusual presentation and successful management, providing valuable insights into the complexities of diagnosing and treating DFSP in atypical presentations.

## Case presentation

Presentation and history

A 59-year-old female presented to our department with a chronic wound on the left shoulder that had lasted for three years. The patient was, otherwise, healthy without a history of previous or current medical conditions. The described lesion started as a small nodule, and because no medical care was sought, the lesion progressed into a wound. Afterward, a primary excision was performed in the dermatology department, and a histopathological exam resulted in a benign lesion. A year later, an open wound occurred at the lesion site that became infected with oozing pus and blood. The patient self-performed wound care by applying dressings. Because the wound remained open and did not heal, the patient came to our clinic seeking medical advice.

Clinical examination

**Figure 1:**
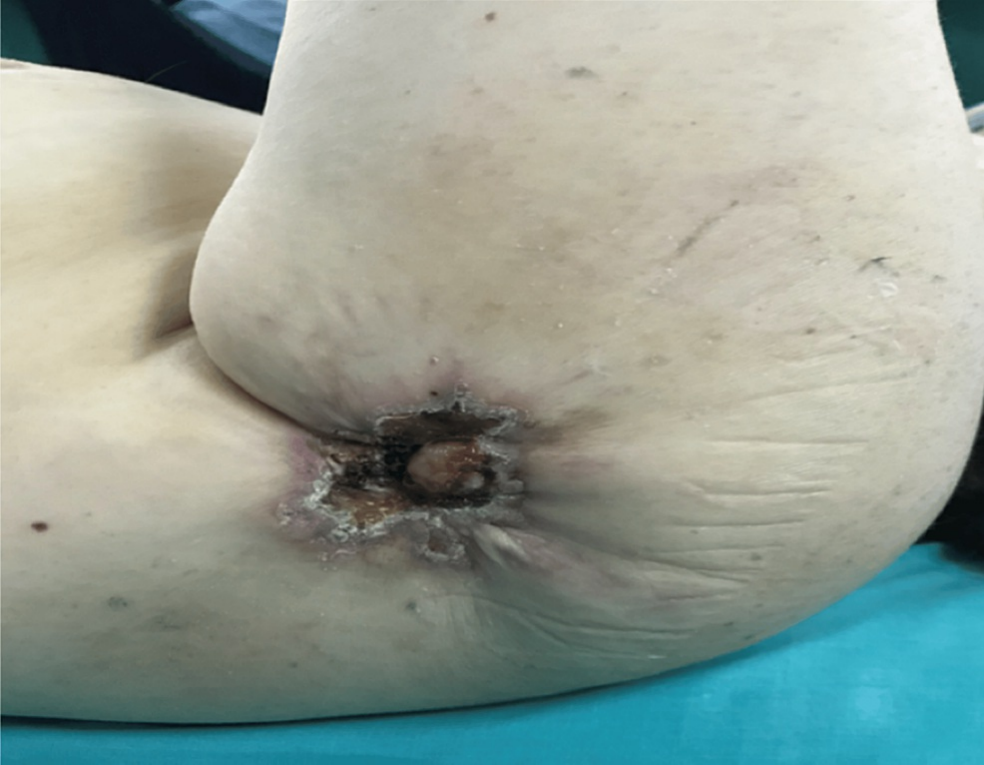
Ulcerative lesion with a contracture scar on the left shoulder

The physical examination revealed an ulcerating lesion with scar contracture in the left shoulder as illustrated in Figure 1. The range of motion of the left shoulder was affected; the patient was unable to fully elevate or abduct the left upper limb.

Management and histopathology

**Figure 2:**
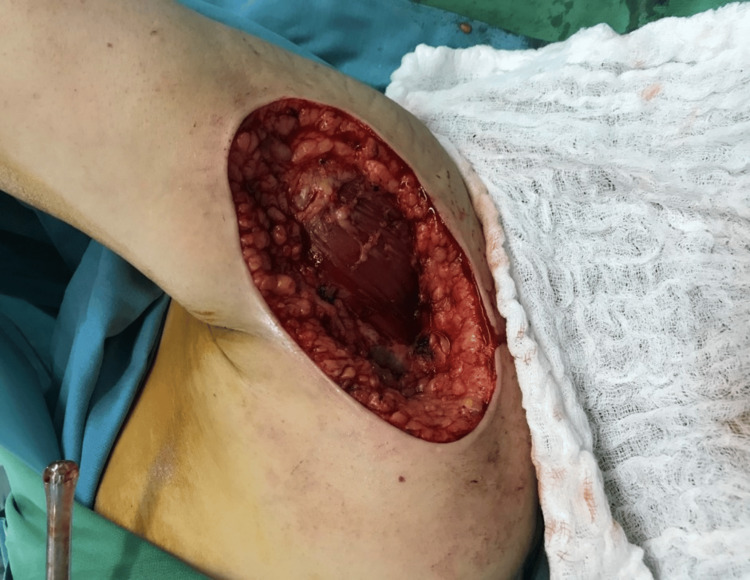
Exposed muscle following a full-thickness excision of the mass

**Figure 3:**
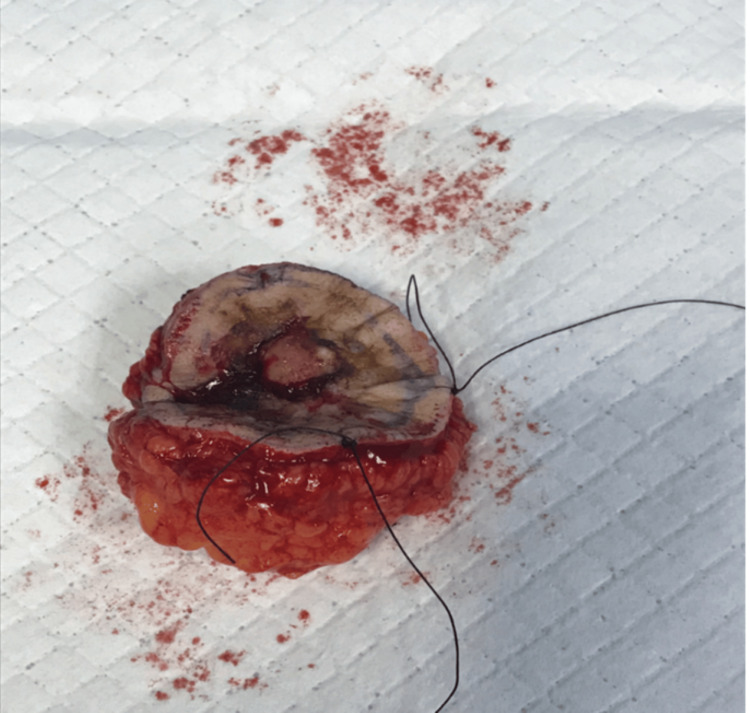
An illustration showing the resected mass (7 x 4 x 2.8 cm)

**Figure 4:**
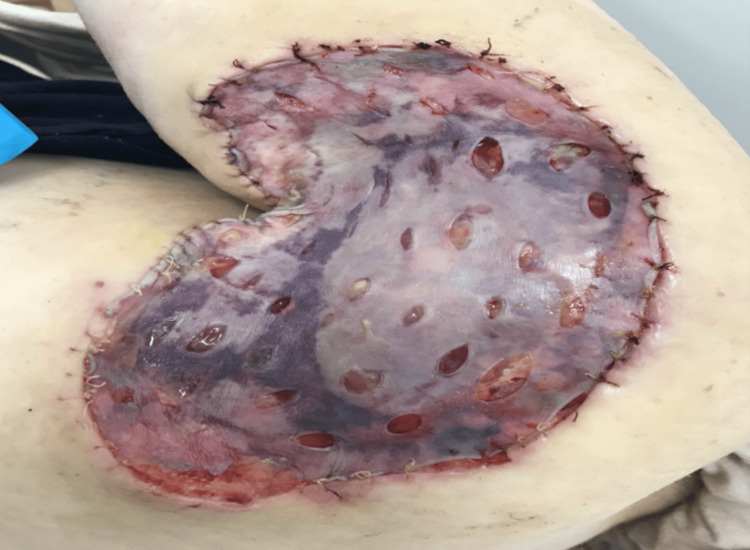
Split-thickness skin graft from the left thigh applied to the lesion site

A decision for excision with a frozen section under general anesthesia was taken. The excision of the left shoulder mass with 1 cm safety margins was done with full-thickness resection (including the fascia) until the muscle layer was exposed (Figure 2). The tumor mass dimensions were 7 x 4 x 2.8 cm (Figure 3), and it was sent to the lab for histopathological assessment during the operation. The frozen section analysis showed negative margins; thus, no additional resection was performed. Then, the defect on the left shoulder was covered with a split-thickness skin graft that was obtained from the left thigh with a dermatome. The graft was sealed with Vicryl 4-0 sutures and fenestration of the graft with blade 15 and dressing with paraffin gauze and bandages was done as shown in Figure 4.

**Figure 5:**
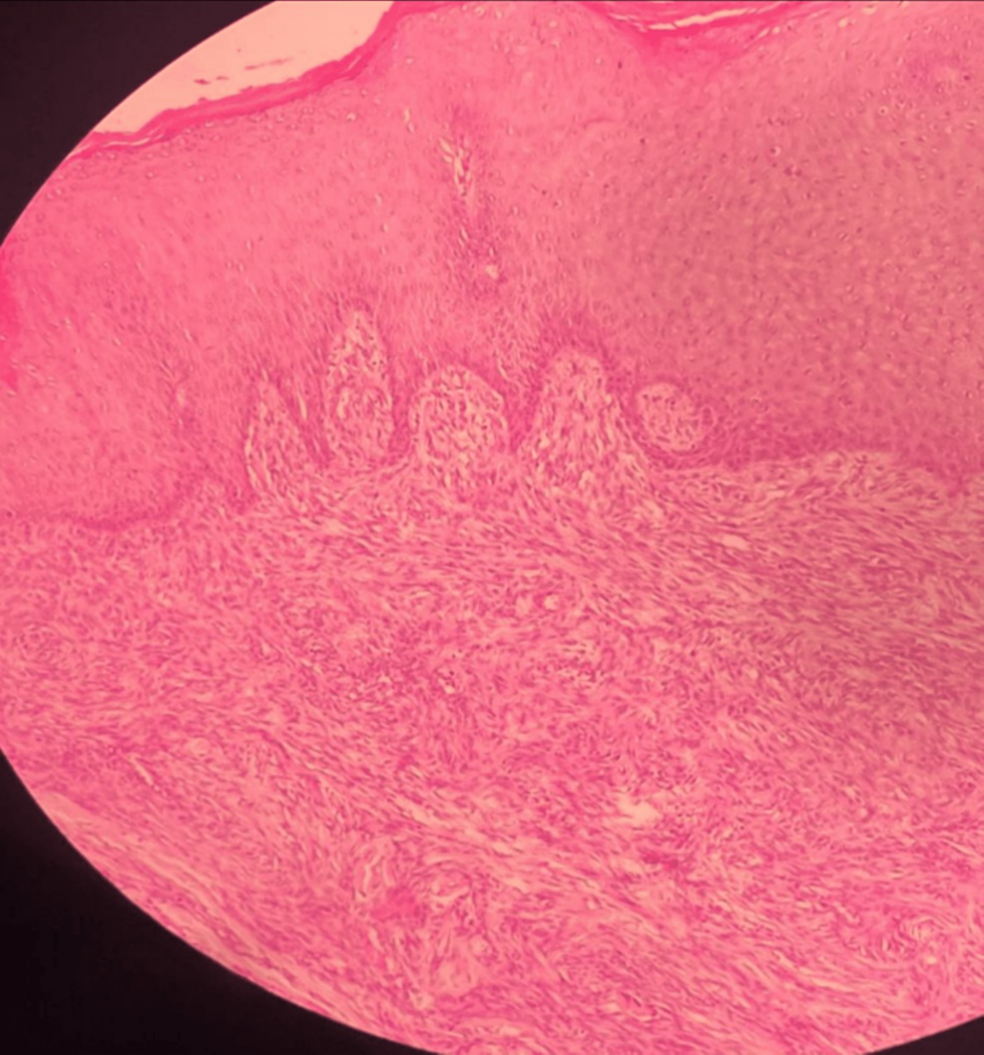
Histopathological examination of the resected mass showing the infiltration to the deep dermis and subcutaneous tissue

**Figure 6:**
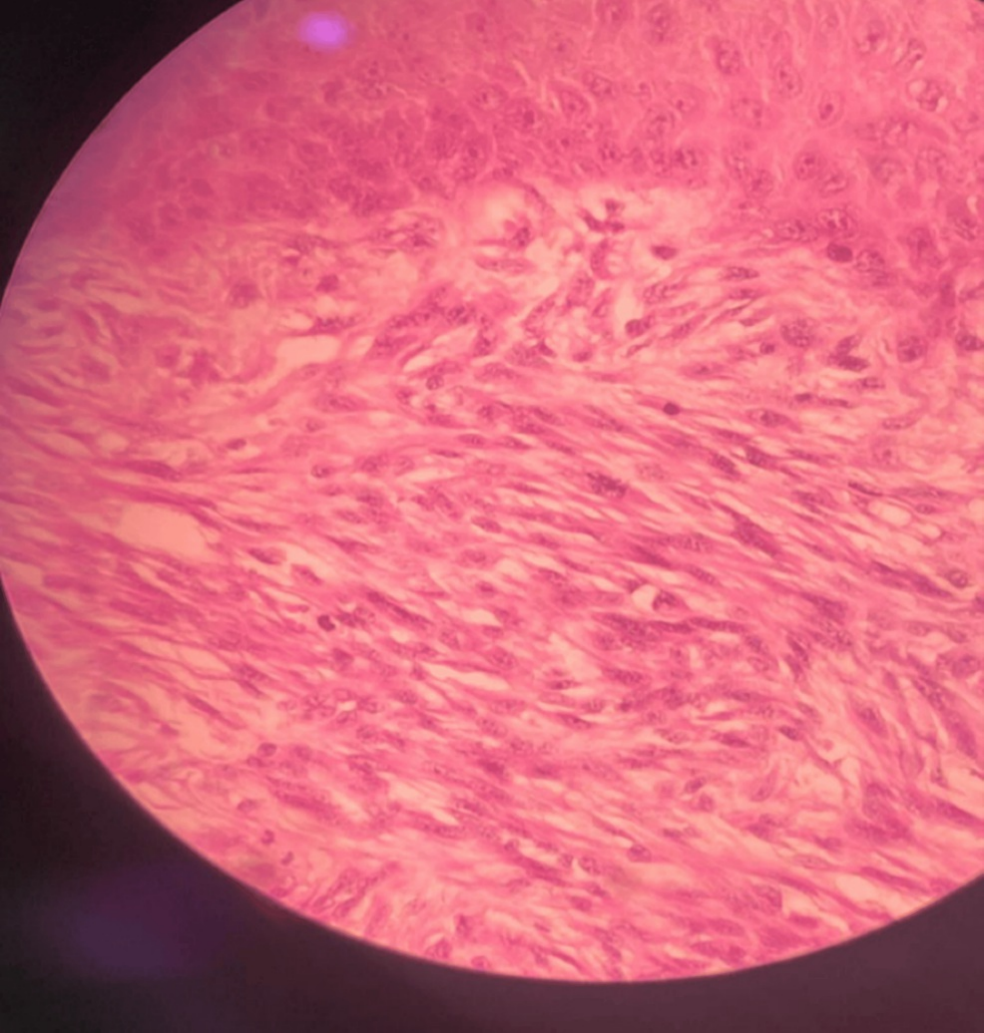
Spindle-shaped fibrous cells consistent with dermatofibrosarcoma protuberans

The histopathological examination revealed a neoplastic lesion consisting of spindle fibrous cells consistent with DFSP. The lesion ulcerated the skin and infiltrated the deep dermal layer and subcutaneous tissue (Figures 5, 6).

Follow up

**Figure 7:**
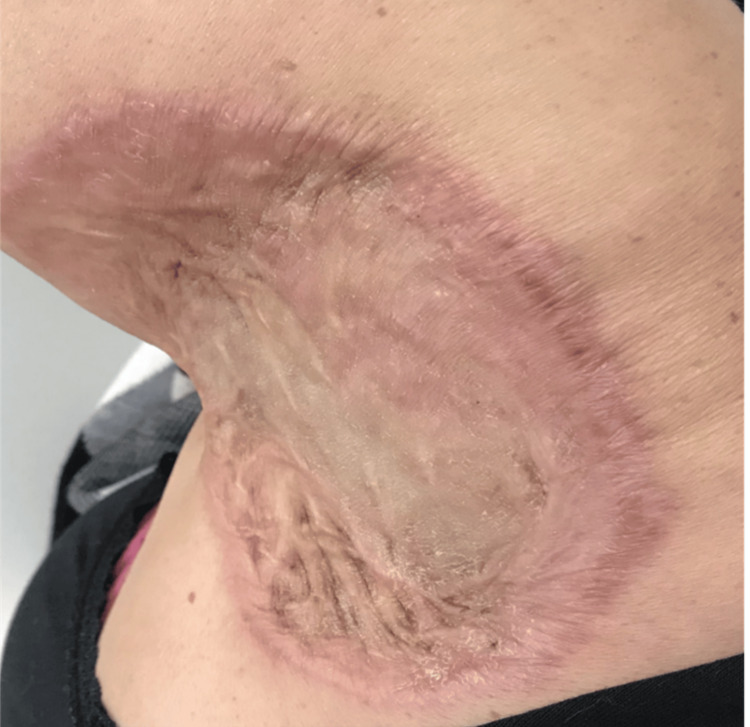
Follow-up image showing the complete healing of the lesion site after grafting

Dressings on the lesion site were changed twice per week, and after two months of follow-up, a complete healing of the graft was observed (Figure 7), and the patient regained full range of motion in the left shoulder.

## Discussion

DFSP is a rare entity, often misdiagnosed due to its indolent nature and resemblance to benign skin conditions. In this case, the patient's DFSP arose from a chronic wound, a presentation scarcely reported in the literature, underscoring the necessity for heightened clinical suspicion in non-healing wounds. Literature reveals that DFSP commonly presents as a cutaneous nodule, with only a handful of cases reported originating from chronic wounds [5]. The rarity of such presentations poses significant diagnostic challenges, as seen in our case, requiring a multidisciplinary approach for accurate diagnosis and management.

The primary management for DFSP is wide surgical excision, considering that DFSP is locally aggressive and widely infiltrative. The principal goal of surgical therapy is the resection of negative margins. Surgical options include standard-wide local excision or Mohs micrographic surgery (MMS), with the choice depending on the individual tumor and the patient's anatomic, functional, and cosmetic considerations [4]. Reconstructive issues should be weighed early in the surgical planning process. A referral to a surgical team with expertise in managing and biologically assessing soft tissue sarcoma is recommended when DFSP is suspected​​ [6].

Historically, DFSP was associated with high local recurrence rates, exceeding 25% in earlier studies where clinical margins were not standardized, and patients rarely underwent adequately wide R0 resection [6]. In contrast, with R0 resection, local recurrence rates have been reported to be between 0.5% and 19%. Wide local excision with margins between 1 cm and 5 cm has shown local recurrence rates ranging from 0% to 11%. The use of 2-cm margins and en-face evaluation of pathologic margins has been reported to result in a local recurrence rate of less than 1%. MMS is particularly valuable when tissue preservation and cosmesis are priorities, such as in head and neck lesions near critical structures [6]. In this case report, safety margins of 1 cm were satisfactory to achieve negative results.

MMS allows for the creation of three-dimensional maps of the lesion and its margins, which can be crucial in achieving clear margins while conserving healthy tissue. Studies have reported no local recurrences at a median follow-up of 38 months in DFSP patients treated with MMS, and similar low recurrence rates in larger patient groups treated with this method​​ [6,7]. The mainstay of DFSP treatment involves surgical excision with clear margins, as inadequate resection is associated with high recurrence rates. Our approach aligns with this protocol, ensuring complete removal of the lesion. Comparative studies suggest that MMS could offer lower recurrence rates, but its application in complex cases like ours might be limited.

Histopathology remains crucial in confirming DFSP, where spindle cell proliferation in a storiform pattern is a hallmark [8]. Our case corroborates this, emphasizing the role of histopathology in guiding treatment. The common histopathological features of DFSP include spindle cells in the deep dermis arranged in a characteristic cartwheel or storiform pattern. These cells often infiltrate adjacent tissues with fingerlike projections. This distinctive pattern is visible using routine hematoxylin and eosin staining. However, DFSP can sometimes be challenging to distinguish from other entities based solely on this staining. Therefore, immunohistochemical (IHC) staining is often employed [2]. A typical staining profile for DFSP includes positive expression of CD34 and vimentin, and the absence of factor XIIIa, which would be positive in dermatofibroma. Additional IHC stains that aid in diagnosis include apolipoprotein, nestin, and cathepsin [8].

DFSP is notorious for local recurrence, warranting vigilant follow-up. A comprehensive analysis of literature from 1996 revealed that the average recurrence rate for DFSP treated with wide local excision was 18% (ranging from 0% to 60%), based on 15 studies. This analysis indicated an overall recurrence rate of 20% in the evaluated cases (100 recurrences out of 489 patients) [9]. On the other hand, an examination of the average recurrence rates from various studies where patients underwent Mohs surgery for DFSP showed a notably lower rate of 0.6% (ranging from 0% to 6.6%). The overall recurrence rate for patients treated with Mohs surgery was found to be 1.6% (1 recurrence in 64 reviewed cases) [9]. In another chart review study, the author observed no recurrences in 63 examined DFSP lesions over a follow-up period of 4.4 years [10]. Excision with negative margins in both primary and recurrent lesions is the main determinant of lower recurrence [1]. Our patient's postoperative recovery, including complete graft healing and restored shoulder mobility, highlights the importance of comprehensive post-surgical care [4]. This case reinforces the need for considering malignancy in chronic non-healing wounds. It also opens avenues for future research in DFSP, particularly in atypical presentations, to enhance understanding and management strategies.

## Conclusions

In conclusion, the presented case of DFSP originating from a chronic wound underscores the diagnostic challenges and therapeutic complexities associated with this rare soft tissue sarcoma. It highlights the importance of maintaining clinical suspicion for malignancy in non-healing wounds, advocating for a multidisciplinary approach for successful management, through wide surgical excision with clear margins and comprehensive post-operative care. Additionally, the case prompts further investigation into optimal management strategies, particularly exploring techniques like MMS. Overall, it contributes to the literature on DFSP, emphasizing the need for continued research to enhance diagnostic accuracy, refine treatment approaches, and ultimately improve patient outcomes.
